# Supplementary material for: Effect of El Niño Southern Oscillation cycle on the potential distribution of cutaneous leishmaniasis vector species in Colombia
Source: PLoS Negl Trop Dis. 2020 May 28;14(5):e0008324. doi: 10.1371/journal.pntd.0008324 (PMC7282671; doi:10.1371/journal.pntd.0008324)
Supplement: S1 Table — (DOCX) [file pntd.0008324.s002.docx]

**Supplementary Table S1** . Episodes of the ENSO cycle between 2007 and 2016 according to the ONI index values of the National Oceanic and Atmospheric Administration

| **Episode** | **Beginning** | **End** | **Months** | **Intensity** |
| --- | --- | --- | --- | --- |
| El Niño 2006-2007 | aug-06 | feb-07 | 7 | Weak |
| Neutral 2007 | mar-07 | may-07 | 3 | N.A. |
| La Niña 2007-2008 | jun-07 | jul-08 | 14 | Strong |
| Neutral 2008-2009 | aug-08 | may-09 | 10 | N.A. |
| El Niño 2009-2010 | jun-09 | may-10 | 12 | Strong |
| La Niña 2010-2011* | jun-10 | jun-11 | 13 | Strong |
| La Niña 2011-2012 | jul-11 | apr-12 | 10 | Moderated |
| Neutral 2012-2015* | may-12 | jan-15 | 33 | N.A. |
| El Niño 2015-2016* | feb-15 | may-16 | 16 | Very strong |
| Neutral 2016 | jun-16 | jun-16 | 1 | N.A. |
| La Niña 2016-2017 | jul-16 | jan-17 | 7 | Weak |

* Episodes used to model changes in the potential distribution of the vectors. N.A.: Not applied
