# Supplementary material for: Effect of El Niño Southern Oscillation cycle on the potential distribution of cutaneous leishmaniasis vector species in Colombia
Source: PLoS Negl Trop Dis. 2020 May 28;14(5):e0008324. doi: 10.1371/journal.pntd.0008324 (PMC7282671; doi:10.1371/journal.pntd.0008324)
Supplement: S2 Table — (DOCX) [file pntd.0008324.s003.docx]

**Supplementary Table S2.** Relationship between occurrence of at least one CL case in each locality and the percentage of local area with a richness of vectors, Norte de Santander (n=1,588 localities)*. PR = Prevalence Ratio.

| **Richness** | **Episode La Niña 2010-2011**  **PR**  **(CI 95%)** | ***p*** | **Episode Neutral 2012-2015**  **PR**  **(CI 95%)** | ***p*** | **Episode El Niño 2015-2016**  **PR**  **(CI 95%)** | ***p*** |
| --- | --- | --- | --- | --- | --- | --- |
| **% no species** | 0.997  (0.982-1.013) | 0.74 | 0.987  (0.976-0.998) | **0.017** | 0.980  (0.950-1.011) | 0.20 |
| **% One specie** | 1.000  (0.991-1.009) | 0.99 | 0.967  (0.942-0.993) | **0.012** | 0.982  (0.966-0.998) | **0.030** |
| **% Two species** | 1.000  (0.992-1.009) | 0.93 | 0.986  (0.980-0.993) | **<0.001** | 0.969  (0.953-0.986) | **<0.001** |
| **% Three species** | 0.999  (0.992-1.006) | 0.70 | 1.007  (1.003-1.011) | **0.001** | 0.989  (0.985-0.993) | **<0.001** |
| **% Four species** | 1.003  (0.994-1.011) | 0.54 | 1.005  (1.002-1.009) | **0.007** | 1.012  (1.008-1.015) | **<0.001** |
| **% <3 species** | 1  (0.994-1.006) | 0.94 | 0.988  (0.983-0.992) | **<0.001** | 0.978  (0.967-0.988) | **<0.001** |
| **% <4 species** | 0.997  (0.989-1.006) | 0.54 | N.A.** |  | 0.988  (0.985-0.992) | **<0.001** |
| **% ≥3 species** | 1.000  (0.994-1.006) | 0.94 | 1.013  (1.008-1.018) | **<0.001** | N.A.*** |  |

* The numbers of localities with at least one case of CL were 172 (10.8%) during the Neutral episode of 2012-2015, 79 (5%) during the La Niña episode of 2010-2011 and 191 (12%) during the El Niño episode of 2015-2016.

**NA: Not applied because the percentages of three and four species are associated with a higher prevalence.

***NA: Not applied because the percentage of three species is associated with a lower prevalence, and the percentage of four species is associated with a higher prevalence.
