# Supplementary figures and images for: Effect of El Niño Southern Oscillation cycle on the potential distribution of cutaneous leishmaniasis vector species in Colombia
Source: PLoS Negl Trop Dis. 2020 May 28;14(5):e0008324. doi: 10.1371/journal.pntd.0008324 (PMC7282671; doi:10.1371/journal.pntd.0008324)

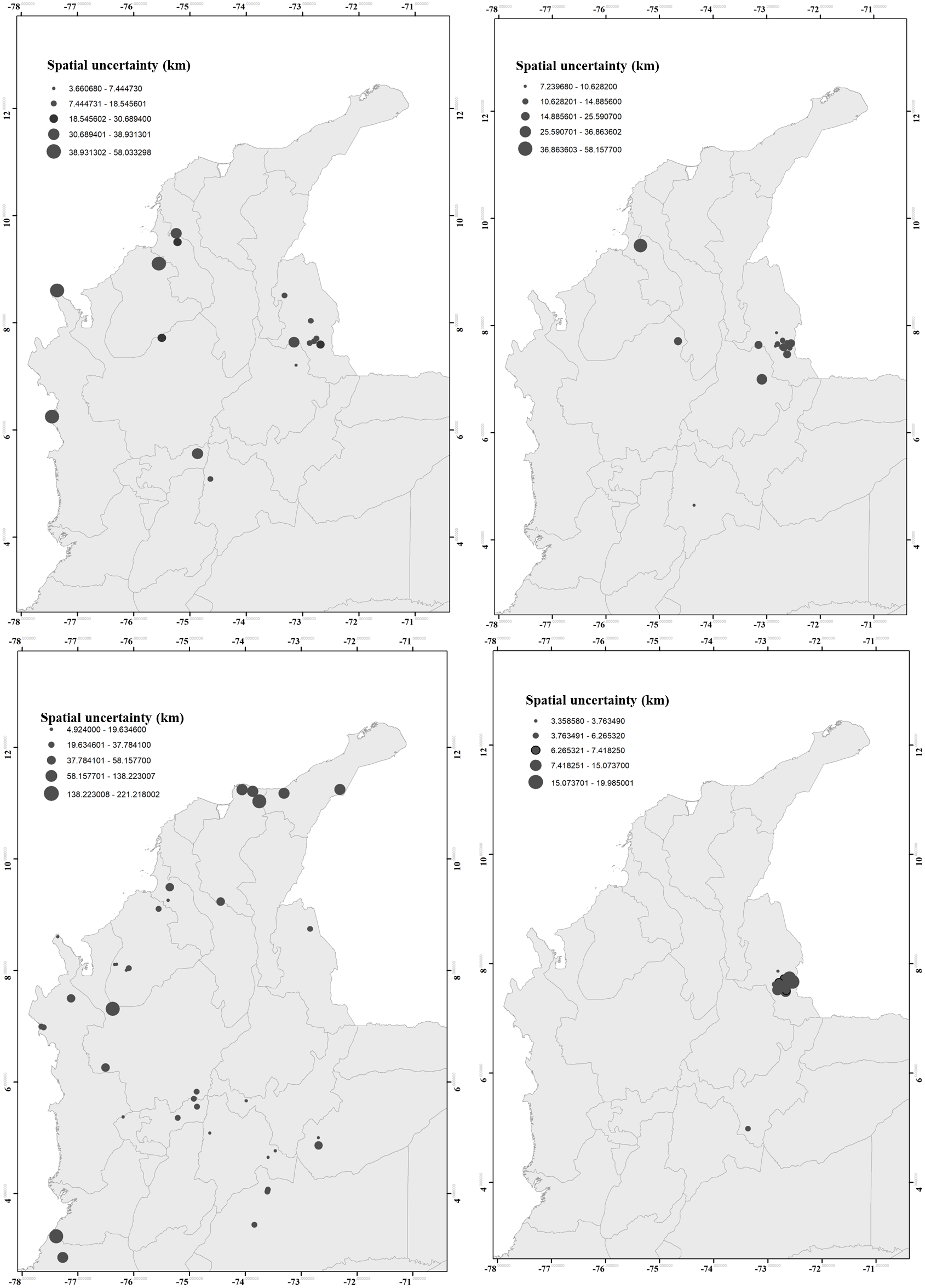

Supplement: S1 Fig — (TIF) [file pntd.0008324.s004.tif]
