# Supplementary material for: Effect of El Niño Southern Oscillation cycle on the potential distribution of cutaneous leishmaniasis vector species in Colombia
Source: PLoS Negl Trop Dis. 2020 May 28;14(5):e0008324. doi: 10.1371/journal.pntd.0008324 (PMC7282671; doi:10.1371/journal.pntd.0008324)
Supplement: Attachment — Submitted filename: Response to Reviwer.pdf [file pntd.0008324.s007.pdf]

Bucaramanga, March 17th, 2020

Doctor:
Hans-Peter Fuehrer
Deputy Editor
PLOS Neglected Tropical Diseases

Dear Dr. Fuehrer,

Thanks for the reviewer comments on our manuscript "Effect of El Niño Southern Oscillation cycle on the potential distribution of cutaneous leishmaniasis vector species in Colombia", PNTD-D-19-02087.

This document containing a detailed list of our responses to the review comments and a description of the changes we have made in the manuscript.

Reviewer's Responses to Questions

Methods

Reviewer #1: Material and methods were properly described and used to achieve the main goals of the study. The limitations of the data were also critically addressed in Discussion section, without belittle the findings.

Reviewer #2: Please see Summary and General Comments

Reviewer #3: The objectives are related to the hypothesis raised.
The approaches used to resolve data inconsistencies or lack of resolution are appropriate.
The sample size is sufficient for the analysis carried out. The variables are relevant but in the case of the land cover layer they use in the different models proposed but nothing is said about it in the Results and discussion section.

R: We understand the reviewer point, but the results section requires a brief functional analysis and we do not mention in a specific way the contribution of any variable. However, in our discussion we have explained how the land use and land cover change could be influencing our results, lines 482-484 "However, these changes were probably mediated by the land cover and the suitability of the habitat for the establishment of viable populations of vectors, particularly in forests [6,44] and perennial crops (e.g., coffee and cocoa)".

Results

Reviewer #1: The data analyses showed the effect of climate changes caused by El Nino in distibution of four vector species of cutaneuous leishmaniasis in Colombia, which are completely new. The Results section is very descriptive, which is a consequence from the type of study.

Minor changes in text and in layout of figures should be considered (details are in comments in the attached pdf).

R: We made the suggested changes in the attached pdf, see bellow.

Reviewer #2: Please see Summary and General Comments

Reviewer #3: The analyses are adequate and the results are quite clear. The figures are adequate and sufficient.
It is recommended that the explanation in the text of the Figures in general should be done in the order in which they appear (A, B, C) and/or be consistent with the models are presented in Materials and Methods: neutral episode, "La Niña" episode and finally "El Niño" episode.

R: We reorganized the next paragraphs:
Lines 403-411 "Of the 1,705 localities identified in Norte de Santander, 40 (2.3%) are urban areas, and 77 (4.5%) are small villages. Among the 1,588 rural localities, during the Neutral episode of 2012-2015 (Fig 6A), 10.8% (172 localities) presented CL cases (372 cases in total, with a median 0 per locality, and ranging from 1 to 24 cases per locality when cases were present); during the La Niña episode of 2010-2011, only 5% (79 localities) had CL cases (131 cases in total, with a median of 0 per locality, and ranging from 1 to 17 cases per locality when cases were present) (Fig 6B); and during El Niño episode of 2015-2016, 12% (191 localities) had CL cases (511 cases in total, with a median of 0 per locality, and ranging from 1 to 19 cases per locality when cases were present) (Fig 6C)."

Lines 431-440 "These statistical findings are related to the observations presented in Figure 7. The intersection between rural localities with potential richness of vectors ≥3 and rural localities with at least one CL case during the Neutral 2012-2015 episode was lower and corresponded to the center zone of the state (Fig 7A). The lowest intersection between rural localities with potential richness of vectors ≥ 3 and rural localities with at least one CL case corresponded to the La Niña 2010-2011 episode (Fig 7B). During the El Niño 2015-2016 episode, nearly the entire state of Norte de Santander (except the northeast region) presented a potential richness of vectors ≥3, and the intersection with rural localities with at least one CL case represented an important extension in the southern and eastern regions of the state (Fig 7C)."

Conclusions

Reviewer #1: The findings is worthy to be published. The discussion section, as well as the Introduction section, are well written and substantiated. The limitations and necessary advances about the study were pointed out.

Reviewer #2: Please see Summary and General Comments

Reviewer #3: The limitations of the work are well delimited and they made relevant approaches to solve them losing spatial resolution but that served to mark a trend.
The main problem in the areas affected by CL is the recording of the case because of the "time span" until the symptoms appear, which makes it difficult to get the date and probable place of infection of this pathology.

R: We agree with the reviewer. We added in lines 524-526 "Likewise, it was difficult to establish the date when the transmission occurred, and the symptom onset date was not available, so the date when the case was recorded by surveillance system was used."

Editorial and Data Presentation Modifications

Reviewer #1: Minor changes are indicated throughout the manuscript file (see attached pdf file).

R: We made the suggested changes in the attached pdf, see bellow.

Reviewer #2: Please see Summary and General Comments

Reviewer #3: “Minor Revision”

Summary and General Comments

Reviewer #1: There is no doubt about how weather conditions affect vector-borne diseases, but it is necessary to study the true impact of the recent climate changes in burden of vector-borne diseases and the authors have collaborated on that, analysing the climate effect in distribution of Lutzomyia vectors on cutaneuous leishmaniasis cases. They report the analyses based on recent El Nino - La Nina cycle, which is a short period, but is worthy to be published, because I considere that small data collection is value to further being part of larger and comprehensive studies.

Reviewer #2: This well-written manuscript presents a model for the effect of El Niño Southern Oscillation (ENSO) on the distribution of vectors and cases of cutaneous leishmaniasis in an endemic region of Colombia. There are some general concerns.

1) Introduction: it is not exactly right that "The effect of the ENSO cycle over the predicted distribution map of the vectors of CL has not yet been assessed, nor has any association of this effect with changes in the occurrence of cases of CL been assessed." (lines 149-151). See, for instance, Yamada et al. (2016, Parasite Epidemiol Control); Ferreira de Souza et al. (2015, Geospat Health); Chaves et al. (2014, PLoS Negl Trop Dis) & Chaves et al. (2008, PLoS Negl Trop Dis).

R: We accepted the suggestion of the reviewer, and we changed the sentence to clarify the idea we want to transmit to the readers, about the novelty of our research (lines 150-153): “In our knowledge, not exist a previous study that simultaneously appraise; a) the effect of the ENSO cycle on the potential distribution of the vectors of CL, and b) evaluate the effect of the changes in the potential distribution of vectors on the occurrence of cases of CL”.

For example, Yamada et al. (2016), Ferreira de Souza et al. (2015) and Chaves et al. (2008) evaluated previously the effect of the ENSO on the incidence of cutaneous leishmaniasis implementing statistical methods as: ANOVA, wavelets and ANCOVA, respectively. Chaves et al. (2014) employed Poisson Rate Generalized Linear Mixed Models to study sand fly abundance patterns across ENSO phases. In all these previous studies the effect of the ENSO cycle over the potential distribution of the vectors of cutaneous leishmaniasis was not assessed, nor the effect of these changes in the potential distribution of vectors with changes in the occurrence of cases of cutaneous leishmaniasis.

2) Methods: I am not sure why you have to choose just one type of each episode (Neutral, La Niña, El Niño) since you have other strong and long events in the period. Their inclusion should add information to the analysis. In any case, the justification that they "corresponded to the most extreme events with the longest duration of their type" (lines 219-220) is not exactly correct

because the first La Niña strong episode (2007-2008) is longer (14, not 13 months) than the one used in the analyses (La Niña 2010-2011, 13 months).

R: We accepted the suggestion of the reviewer. In that sense, we modified the Supplementary_Table_1 because the episode of La Niña 2010-2011 started in May 2010 (not in June) and finished in June 2011. Additionally, we modified the lines 222-226 to explain why we excluded the episode of La Niña 2007-2008, lines 222-226 "We excluded the episode of La Niña 2007-2008 because according to the Institute of Health of Norte de Santander the 2007 report of CL cases was inaccurately documented by the surveillance system (mainly in rural areas); this phenomenon could correspond to a conditioning period in the healthcare institutions."

3)Methods: please provide some uncertainty measure for pROC (e.g., minimum and maximum based on bootstrapping). The problem of partial ROC analysis to test statistical significance of ecological niche model predictions is that it does not provide a measure of how much good is the prediction, you just know that 1.0 corresponds to a random classifier, so you can test significance but this information which is too vague to assess the quality of the prediction. Even knowing the limitations of the regular AUC analysis in such a setting, I would like to see the % of correct classifications for presence points and for a random sample of absence points, and also the number of wrong presence predictions.

R: This was a useful suggestion. Now we have included in the results the edited table including the maximum and minimum values, correct classification rate and omission error (fraction). See Table in line 347.

4) Methods: The authors used a random sample of 30% of the distribution data to evaluate the model. Please make sure to give a detailed description of this process. Is the 30% used to test and validation was done in the other 70%? Did you use some cross-validation? Is the error greater or lower for points located in the state of Norte de Santander as compared to points outside?.

R: As recommended, technical details on the methodology were modified and we included in line 291-292 "…and occurrence data for each species was split into training (70%) and evaluation (30%).".
We didn't use cross-validation. On the other hand, is important to clarify that the calibration of the model was carried out in the M and was transferred and evaluated in the entire study area, the political division the state of Norte de Santander was just used to evaluate the relationship between the potential richness of the vectors and CL occurrence.

5) Discussion: should highlight problems in prediction based on incomplete data (only those that could be assessed -- publication bias; mainly presence data - misclassification bias; data not well distributed in the area, etc.).

R: We accepted the suggestion of the reviewer, and we modified the lines 536-538 to include the bias mentioned by the reviewer. "For last, we recognize gaps in the prediction of potential distribution of CL vectors, based on incomplete data; publication, taxonomic, and misdetermination bias; and not well distributed data in the country."

Reviewer #3: In general the study is well supported by the data it uses and provides in the supplements. Some minor revisions and further details are highlighted and requested in the manuscript.

Highlighted and requested in the manuscript PDF.

a. **REVIEWER (1)**

Line 204-208: It is explained why the reclassified coverage layer was included, but nothing is said about its influence on results and discussion.

R: We understand the reviewer point, but the results section requires a brief functional analysis and we do not mention in a specific way the contribution of any variable. However, in our discussion we have explained how the land use and land cover change could be influencing our results, lines 482-484 “However, these changes were probably mediated by the land cover and the suitability of the habitat for the establishment of viable populations of vectors, particularly in forests [6,44] and perennial crops (e.g., coffee and cocoa)”.

Line 213: In Supplement 1 reference (.docx) you should correct: "Supplementary Table S2" by "Supplementary Table S1".

R: We changed it.

Line 260-261: names lowercase in all cases.

R: We changed it.

Line 308-311: What was the criterion used to characterize urban, suburban and rural areas?

R: We accepted the suggestion of the reviewer. In that sense, we clarified in lines 314-317 “ … because of the uncertainty about the location where the infection occurred in the cases reported in urban areas and small villages and the low number of cases reported in such areas. This administrative division is based on the national surveillance system.”

Line 427-428: reference (.docx) you should correct: "Supplementary Table S3" by "Supplementary Table S2"

R: We changed it.

b. **REVIWER**

Line 76: two vector species?

R: We corrected the sentence. Line 77 “two vector species increased their distribution into environmentally suitable areas,..”

Line 90: Reference

R: We corrected the sentence. Lines 90-91 “More than 70% of all CL cases worldwide occur in only 10 countries, including Colombia [2].”

Line 152: four vector species

R: We corrected the sentence. Line 153-154 “…the potential distribution of four vector species of CL…”

Line 165: insert " " quotation marks

R: We corrected the sentence. Line 166 "…the terms "cutaneous" and "leishmaniasis" and "Colombia" and "Lutzomyia"…"

Line 199:> 0.8? Not < 0.8 instead?

R: We corrected the sentence. Line 200 "…variables with correlation value >|0.8| were removed."

Line 346: Reorganize the mentioning of figures, is not ordened... or reorganize the layout of figures 1-4. (Frase introductoria de las figuras para citarlas)

R: We added in line 352 the sentence "The potential distribution of the four Lutzomyia species during the Neutral 2012-2015, the La Niña 2010-2011, and the El Niño 2015-2016 episodes are shown in the figures 1 to 4."

Line 357: For better conexion between description results and images, I suggest combine the 4 figures of potential distribution maps in one, designating A to D for each species, and inserting climate episodes as headers in the image maps

R: We accepted the suggestion of the reviewer of inserting climate episodes as headers in the image maps, and we modified the figures 1-4. However, we consider that combine the 4 figures (12 images in total) could make difficult the view and the interpretation of the information.

Line 391: Legends tend to be very repeatitive. Insert headers regarding climate episodes for better understanding and to avoid repetitive descriptions. La Nina as top header for maps at left panels and El Nino as top header for right panel; thus will avoid every repeat.

R: We inserted headers regarding climate episodes in figures 1 to 5, also we simplified the legends of these figures. Lines 365 – 387 and 395 – 399.

Line 392: each Lutzomyia vector.

R: We corrected the sentence. Lines 395-396 "Fig 5. Environmental similarity estimated by MOP metric between M and transference zone for each *Lutzomyia* vector."

Line 405 order of results' description shoud follows alphabetical order of the layout (position) of figures; start with A and so on.

R: We reorganized the paragraph:
Lines 403-411 "Of the 1,705 localities identified in Norte de Santander, 40 (2.3%) are urban areas, and 77 (4.5%) are small villages. Among the 1,588 rural localities, during the Neutral episode of 2012-2015 (Fig 6A), 10.8% (172 localities) presented CL cases (372 cases in total, with a median 0 per locality, and ranging from 1 to 24 cases per locality when cases were present); during the La Niña episode of 2010-2011, only 5% (79 localities) had CL cases (131 cases in total, with a median of 0 per locality, and ranging from 1 to 17 cases per locality when cases were present) (Fig 6B); and during El Niño episode of 2015-2016, 12% (191 localities) had CL cases (511 cases in total, with a median of 0 per locality, and ranging from 1 to 19 cases per locality when cases were present) (Fig 6C)."

Line 412: Insert a map of Colombia to position the Norte de Santander region and better integration with previous figures (as done in figure 7).

R: We accepted the suggestion of the reviewer, and we modified the figure 6 including a map of Colombia to position the Norte de Santander state.

Line 431: move this sentence to end of paragraph, to follow the order of figure A > B > C.

R: We reorganized the paragraph:
Lines 431-440 “These statistical findings are related to the observations presented in Figure 7. The intersection between rural localities with potential richness of vectors ≥3 and rural localities with at least one CL case during the Neutral 2012-2015 episode was lower and corresponded to the center zone of the state (Fig 7A). The lowest intersection between rural localities with potential richness of vectors ≥ 3 and rural localities with at least one CL case corresponded to the La Niña 2010-2011 episode (Fig 7B). During the El Niño 2015-2016 episode, nearly the entire state of Norte de Santander (except the northeast region) presented a potential richness of vectors ≥3, and the intersection with rural localities with at least one CL case represented an important extension in the southern and eastern regions of the state (Fig 7C).”

Line 453: Please, clarify and state here, the difference between your study and that published by Chaves LF et al Plos NTD PLoS Negl Trop Dis, 8 (10), e3210, 2014. Reference 11.

R: We accepted the suggestion of the reviewer and changed the sentence in line 454-457, to clarify the novelty of our research “However, to our knowledge, this is the first study that implements niche modeling to predict the change in the potential distribution of CL vectors associated with the episodes of the ENSO cycle. Additionally, it is the first to evaluate if these changes in the potential distribution impact the occurrence of cases of CL.”
In the case of the study published by Chaves et al. (2014). They employed Generalized Linear Mixed Models to study vectors’ abundance across ENSO phases. However, in our paper we implemented niche modeling to assess the effect of the ENSO cycle on the potential distribution of the vectors of CL, and we contrasted the effect of these changes in the potential distribution of vectors with changes in the occurrence of cases.

Sincerely,

Ruth Martínez-Vega
